# Comparison of sexual function of people with colorectal cancer with and without colostomy bag in Iran: a comparative cross-sectional study

**DOI:** 10.1038/s41598-023-39728-9

**Published:** 2023-08-02

**Authors:** Amirmohammad Dahouri, Mohammad Hassan Sahebihagh, Neda Gilani

**Affiliations:** 1 Departement of Community Health Nursing, Faculty of Nursing and Midwifery, Tabriz University of Medical Sciences, Tabriz, Iran; 2 Department of Statistics and Epidemiology, Faculty of Health, Tabriz University of Medical Sciences, Tabriz, Iran

**Keywords:** Cancer, Psychology, Gastroenterology, Health care, Oncology

## Abstract

The aim of this study was to investigate and compare sexual function in individuals with colorectal cancer, with and without a colostomy bag. A quantitative, descriptive-comparative design was employed, and a cluster random sampling method was used to recruit 252 patients with colorectal cancer. Data collection tools included a participants characteristics form, the International Index of Erectile Function (IIEF) for men, and the Female Sexual Function Index (FSFI) for women. The mean IIEF total score for men with a colostomy was 26.17 ± 15.30, and for men without a colostomy, it was 29.05 ± 17.14. The mean FSFI total score for women with a colostomy was 6.40 ± 7.21, and for women without a colostomy, it was 9.10 ± 14.67. There was no statistically difference in IIEF scores between men with and without colostomy bags (*p* > 0.05). However, women with colostomy bags had significantly lower FSFI scores compared to women without colostomy bags (*p* < 0.05). Addressing sexual concerns in individuals with colorectal cancer is crucial for enhancing their sexual well-being and overall quality of life. Comprehensive support, timely interventions, and targeted services are essential to help patients navigate the challenges and improve their overall well-being.

## Introduction

Cancer comprises a diverse group of diseases that are unique to multicellular organisms, characterized by uncontrolled cellular proliferation and growth^1^. It poses a significant global health challenge and stands as the second leading cause of mortality in the United States^2^. Annually, approximately 30,000 individuals lose their lives to cancer^3^. Colorectal cancer (CRC) is a widespread malignancy worldwide, with projections indicating a substantial increase in its incidence, reaching an estimated 3.2 million new cases and 1.6 million deaths by 2040^4^. Developed countries account for over half (55%) of these cases^5^. In Iran, the incidence rate of CRC has witnessed a marked rise over the past 25 years^6^. Reports indicate that CRC ranks as the fourth most common cancer in Iran, ranking third among Iranian women and fifth among Iranian men^7^. Regrettably, CRC claims the lives of approximately 30,000 individuals in Iran each year^3, 8^. The escalating incidence of CRC can be attributed to factors such as increased life expectancy, changes in lifestyle patterns, and advancements in diagnostic and therapeutic approaches^9^. CRC exerts profound implications on both individual well-being and the socioeconomic fabric of societies^10^. A study assessing the economic burden of CRC revealed the estimated follow-up treatment costs to be 2220 euros for colon cancer and 4801 euros for rectal cancer^11^. Furthermore, individuals diagnosed with CRC often experience a range of distressing symptoms, including weakness, fatigue, cognitive impairment, loss of appetite, nausea, vomiting, anemia, and nutritional deficiencies^12, 13^.

Furthermore, there has been a significant increase in the population of individuals with a history of colorectal cancer (CRC) owing to remarkable advancements in treatment. This includes those who have undergone permanent ostomy procedures, redirecting feces and urine through a surgically created opening in the abdominal wall^14, 15^. Annually, more than 70,000 individuals in England and nearly 120,000 individuals in the United States undergo ostomy surgeries^16, 17^. Sexual problems exhibit a high prevalence among cancer patients, encompassing prostate, female, breast, head and neck, blood, and colorectal cancers, with reported rates ranging from 53 to 91%^18, 19^. Survivors of CRC who engage in sexual activities often encounter challenges in sexual performance, attributed to factors such as therapeutic surgeries, radiotherapy, or the presence of an ostomy^20^. Moreover, a study by Dulskas et al. (2016) reveals that sexual and urinary problems commonly manifest following rectal cancer surgery, with a particularly elevated prevalence of dysfunction observed among female patients^21^.

Given the increasing incidence of colorectal cancer (CRC) and its profound implications, there is a critical need to explore the challenges faced by CRC patients in terms of their sexual function. However, limited research exists on the specific impact of colostomy bags on sexual function, indicating a significant gap in our understanding. Therefore, this study aims to comprehensively compare the sexual performance of CRC patients with and without colostomy bags in Iran. By shedding light on this crucial aspect of CRC survivorship, the study seeks to provide valuable insights and contribute to the existing knowledge regarding the association between colostomy and sexual function in the context of CRC.

## Methods

### Ethical consideration

In this study, strict adherence to ethical principles was ensured, and all necessary approvals and permissions were obtained from the relevant authorities. The research plan received approval from the Research Council and the Research Vice-Chancellor of the Faculty of Nursing and Midwifery at Tabriz University. Furthermore, permission to conduct the study was granted by the esteemed Research Vice-Chancellor of Tabriz University of Medical Sciences, with the regional ethics committee providing approval under reference number IR.TBZMED.REC.1401.046. Prior to commencing the study, permission was obtained from the research environment where the investigation took place. To ensure the protection of participants' rights and privacy, all potential participants were provided with a clear explanation of the research objectives, and their voluntary participation was sought. Confidentiality was safeguarded by assuring participants that their personal information would be treated with the utmost confidentiality. Anonymity was maintained by utilizing a coding system instead of using actual names in the questionnaire. The study adhered to ethical guidelines regarding the use of other research and sources, employing proper citation and referencing to acknowledge the original authors and respect intellectual property rights. Moreover, in the interest of transparency and accountability, the research findings were made available to the participants upon request. By upholding these ethical considerations, the study aimed to protect the rights and well-being of the participants, ensure the confidentiality of their information, and maintain the integrity and reliability of the research findings.

### Study design

A comparative, cross-sectional design was adopted to identify and compare sexual function in CRC patients with and without colostomy bag.

### Instruments with validity and reliability

#### Participants' characteristics

In this study, the characteristics of the patients were assessed through a researcher-designed questionnaire. The questionnaire aimed to capture various demographic and clinical variables.

#### Women's sexual performance index (FSFI) questionnaire

In order to evaluate sexual function among female participants, the Female Sexual Function Index (FSFI) was administered. The FSFI consists of 19 questions that assess six domains of sexual function, including desire, arousal, moisture, orgasm, satisfaction, and pain. The assessment was conducted using a Likert scale. The total score was calculated by summing the scores from each domain. The reliability and validity of this questionnaire have been confirmed in Iran^22–25^.

#### International index of erectile function in men questionnaire (IIEF)

The International Index of Male Erectile Function (IIEF) Questionnaire is utilized as a tool to measure the sexual performance of men. It consists of 15 questions that assess various aspects of erectile function across five distinct subscales. Notably, the scores obtained from this questionnaire are standardized by the researcher, and no specific threshold is set for interpretation. Also, it should be noted that the reliability and validity of this questionnaire have been confirmed in Iran^26, 27^.

### Sampling and recruitment

#### Sample size and power

In this research study, the quality of life scores (SF-36) were examined in two distinct groups of patients diagnosed with CRC: those with a colostomy bag (mean score: 74.1 ± 6.3) and those without a colostomy bag (mean score: 80.3 ± 8.2) based on the findings of Näsvall et al.'s study^28^. To determine the required sample size, considering an alpha level of 0.05, a power of 0.9, and employing the formula for calculating the mean difference in two independent populations ${{\varvec{n}}}^{\mathbf{*}}=\frac{{\left({{\varvec{Z}}}_{1-\boldsymbol{\alpha }/2}+{{\varvec{Z}}}_{1-{\varvec{\beta}}}\right)}^{2}({{\varvec{S}}}_{1}^{2}+{{\varvec{S}}}_{2}^{2})}{{{\varvec{d}}}^{2}}$, a sample size of 25 participants was estimated. In addition, the sample size for this study was determined considering cluster random sampling, a design effect coefficient of 2, and an anticipated dropout rate of 20 percent. Based on these factors, the final sample comprised 126 patients, with 63 female patients in the colostomy group (referred to as the women case group), 63 female patients without a colostomy bag, 63 male patients in the colostomy group, and 63 male patients without a colostomy bag. It is important to note that precise calculations for the sample volume were conducted using the G-power software, ensuring accuracy in the determination of the sample size^29^.

### Population and sample

The target population of this study comprised individuals diagnosed with CRC, irrespective of whether they had a colostomy bag or not, and regardless of the bag's permanence or temporariness.

#### Data collection

The study commenced by obtaining the necessary permissions from the Research Vice-Chancellor of Tabriz University of Medical Sciences. Following this, the researcher sought approval from the Vice-Chancellor of the University to conduct research on patients. Subsequently, the researcher approached the heads of relevant hospitals, presenting a letter of introduction to obtain permission for conducting the research. Coordination with the supervisors of the chemotherapy departments was established, and the researcher scheduled visits to the hospitals, ensuring minimal disruption to patient care. The hospitals included Shahid Madani, Shahid Ghazi, Ali Nasab, Shahriar, and Valiasr in Tabriz. Upon arrival at the chemotherapy outpatient departments, eligible and interested individuals were invited to participate in the study and complete the research questionnaire. The researcher introduced themselves to the patients at their bedside, providing a clear explanation of the research objectives, emphasizing voluntary participation, and assuring confidentiality of participants' information. The questionnaires were then distributed to the patients for completion. Throughout the questionnaire completion process, the researcher remained present to assist patients and address any queries or concerns, ensuring accurate and error-free completion of the questionnaires. It is worth noting that prior to the patients beginning the questionnaire, a concise explanation was provided on how to properly complete it. The completed questionnaires were collected during the same session. Data collection took place between April 1, 2022, and May 1, 2022. It is imperative to acknowledge that cluster random sampling was utilized as the sampling method in this study.

#### Data abstraction

##### Entry criteria

A confirmed diagnosis of CRC by an oncologist, presence of a colostomy bag (either permanent or temporary), ability to effectively communicate, willingness to participate in the study, age over 30 years, referral for outpatient chemotherapy, knowledge about their illness and treatment, active engagement in sexual performance, and a history of sexual function prior to the onset of the disease.

##### Exclusion criteria

**Figure 1:**
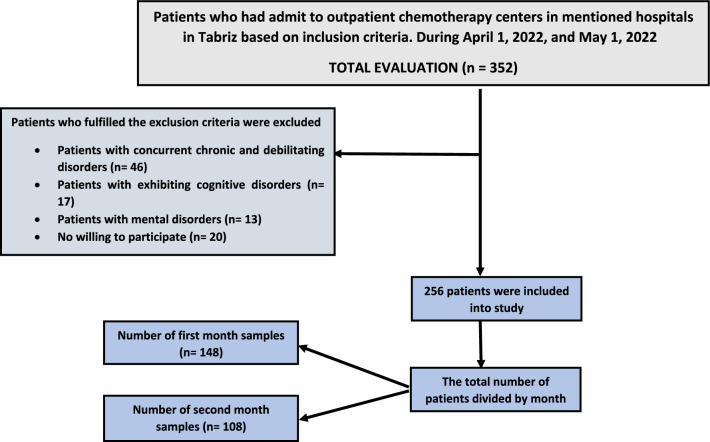
Consort flow diagram (Inclusion–Exclusion criteria).

Participants with concurrent chronic and debilitating diseases, including diabetes, kidney diseases, or any organ defects that could potentially impact sexual function, as reported by the participants, were excluded. Additionally, individuals exhibiting cognitive disorders, such as Alzheimer's disease, as reported by the participants themselves or their companions, were not included. Known mental disorders, either reported by the participants or documented in their medical records, served as additional exclusion criteria. Furthermore, individuals who expressed unwillingness to participate in the study were excluded from the sample. These stringent criteria aimed to enhance the homogeneity of the participant group and minimize confounding factors that could influence the study outcomes. It should be noted that Fig. 1 shows the sampling flow diagram which provides a comprehensive overview of the participants selection process.

#### Data analysis

The data analysis was performed using IBM SPSS Statistics version 24. To analyze the demographic information of the samples, frequency and percentage distributions were used. Descriptive statistics, such as mean and standard deviation, were employed to summarize the sexual function variable, which exhibited normality. The normality assumption of the variable distribution was assessed through the Kolmogorov–Smirnov test, along with Skewness and Kurtosis indices. A significance level of 0.05 was considered for all statistical tests conducted in this study. For qualitative variables, the Chi-Square test was utilized to compare two groups, provided that the Cochrane conditions were satisfied. In cases where the Cochrane conditions were not met, the Chi-Square test based on Monte-Carlo Simulation was employed to compare the two groups when the variable in question was quantitative and normally distributed. When conducting quantitative comparisons of sexual function scores, the independent samples t-test was used, while the chi-test was employed for qualitative comparisons. To control for potential confounding factors, general linear modeling and multiple regression techniques were employed. These statistical methods allow for the assessment of confounder effects and enhance the validity and reliability of the study's findings.

### Ethics approval and consent to participate

Ethical approval for the study was granted by Tabriz University of Medical Sciences with ID: IR.TBZMED.REC.1401.046. (the research ethics committees certificate file has been uploaded in the supplementary section). Informed consent was obtained from all participants. There were no under 16 participants in this research. All methods were carried out in accordance with relevant guidelines and regulations.

## Results

Table 1 provides the participants’ characteristics of the study. A total of 256 colorectal cancer patients participated, with 129 males (49.6% with a colostomy bag, 51.2% without) and 127 females (50.4% with a colostomy bag, 48.8% without). The majority were married (206 individuals). The age range with the highest frequency was 30–40 years (67 individuals), while the lowest frequency was seen in those over 60 years (28 individuals). Education-wise, 81 had a bachelor's degree, and 50 had a diploma. Employment was reported by 166 participants. Income matching expenses was stated by 138 individuals. Insurance coverage was reported by 228 participants. The duration since diagnosis varied, with 17.6% reporting ten months. Most participants were city residents (240 individuals), with 228 living in their own homes. Treatment combinations included only chemotherapy (81 patients), chemotherapy, radiotherapy, and surgery (98 patients), and chemotherapy with surgery (77 patients). Regarding the time since the last chemotherapy session, 232 participants completed the questionnaire after two weeks. A family history of cancer was positive for 121 patients, while metastasis was observed in 149 cases. The majority of participants had undergone 1–10 chemotherapy courses (137 patients) and had at least one additional disease besides cancer (106 patients). The time since the last surgery ranged from 1 to 10 months for most participants (223 individuals). Less than 10 h of sports activities per week were reported by 229 participants. Sexual activity prior to the disease was stated by 251 participants. Weight categories included 63 patients weighing 65–45 kg, 126 weighing 85–65 kg, and 67 weighing 105–85 kg. The majority of participants fell within the height range of 192–130 cm (253 individuals), and the most common body mass index range was 18.5–25 (123 individuals).

**Table 1:** Distribution of frequency and percentage of individual social characteristics of samples and comparison of classes.

Variable	Classes	N (valid percent)
Age	30 to 40	67 (26.2)
	40 to 50	46 (18.0)
	50 to 60	95 (37.1)
	More than 60	48 (18.8)
Sex	Male	129 (50.4)
	Female	127 (49.6)
Marital status	Single	22 (8.6)
	Married	206 (80.5)
	Divorced and widowed	28 (10.9)
Having child	Yes	196 (76.6)
	No	60 (23.4)
Education	Under Diploma	50 (19.5)
	Diploma	73 (28.5)
	Bachelor	81 (31.6)
	Post Graduate	52 (20.3)
Job	Employed	166 (64.8)
	Unemployed	90 (35.2)
Income adequacy	Income Equals Expenditure	138 (53.9)
	Income More Than Expenditure	42 (16.4)
	Income Less Than Expenditure	76 (29.7)
Having insurance	Yes	228 (89.1)
	No	28 (10.9)
Location	City	240 (93.8)
	Village	16 (6.3)
Housing type	Personal	228 (89.1)
	Rent	28 (10.9)
Type of treatment	Only Chemotherapy	81 (31.6)
	Chemotherapy–Radiotherapy-surgery	98 (38.3)
	Chemotherapy-surgery	77 (30.1)
Time of last chemotherapy (week)	< 5	232 (90.6)
	≥ 5–10	24 (9.4)
	Mean (SD)	3.10 (4.04)
Family history	Positive	121 (47.3)
	Negative	135 (52.7)
Metastasis	Yes	149 (58.2)
	No	107 (41.8)
Number of chemotherapy courses (Number)	< 10	137 (53.5)
	≥ 10–20	84 (32.8)
	≥ 20–30	35 (13.7)
	Mean (SD)	9.34 (6.98)
Another disease besides cancer	Yes	106 (41.4)
	No	150 (58.6)
Time of last surgery (Month)	1–10 ≥	223 (87.1)
	11–20 And more	33 (12.9)
	Mean (SD)	6.11 (5.52)
Exercise (Hour/Week)	≤ 10	229 (89.5)
	10–20 And more	27 (10.5)
	Mean (SD)	3.42 (3.84)
Sexually active before the disease	Active	251 (98)
	Not Active	5 (2)
Weight (kg)	45–65	63 (24.6)
	≥ 65–85	126 (49.2)
	≥ 85–105	67 (26.2)
	Mean (SD)	74.71 (14.42)
Height (cm)	70–130	3 (1.2)
	≥ 130–192	253 (98.8)
	Mean (SD)	169.34 (13.48)
Body mass index (kg/m^2^)	< 18.5	12 (4.7)
	≥ 18.5–25	123 (48)
	≥ 25–30	67 (26.2)
	≥ 30	54 (21.1)
	Mean (SD)	25.88 (4.74)
Having colostomy	With	127 (49.6)
	Without	129 (50.4)
Time of detection (Month)	Mean (SD)	14.92 (7.50)

Table 2 displays the results of the statistical analysis conducted on the FSFI questionnaire, which assessed women's sexual performance across six domains. Comparing women with and without colostomy bag, significant differences were found in all domains (*p* < 0.05). Women without bags had higher average scores (9.10 ± 14.67) compared to women with bags (6.40 ± 7.21). Notably, among women without colostomy bag, 61 (96.8%) achieved poor performance (28 ≥), while all 64 (100.0%) women with colostomy bag exhibited poor performance (28 ≥).

**Table 2:** Distribution of frequency, percentage, the mean and standard deviation of female sexual function index (FSFI) and comparing the scores separately for having and not having a colostomy bag (n = 127).

Variable	Classes (Cut of Points)	Female with colostomy N (%)	Female without colostomy N (%)	*p*-value (Qualitative)	*p*-value (Quantitative)
Sexual desire	Desirable performance (> 3/3)	6 (9.4)	22 (34.9)	0.001*	< 0.001***
	Poor performance (≤ 3/3)	58 (90.6)	41 (65.1)		
	Total	64 (100.0)	63 (100.0)		
	Mean (SD)	1.72 (0.92)	2.66 (1.34)		
Mental stimulation	Desirable performance (> 4/3)	2 (3.1)	8 (13.1)	0.051**	0.001***
	Poor performance (≤ 4/3)	62 (96.9)	53 (86.9)		
	Total	64 (100.0)	63 (100.0)		
	Mean (SD)	1.72 (0.60)	2.38 (1.41)		
Humidity	Desirable performance (> 4/3)	6 (9.4)	25 (39.7)	0.001*	< 0.001***
	Poor performance (≤ 4/.3)	58 (90.6)	38 (60.3)		
	Total	63 (100.0)	64 (100.0)		
	Mean (SD)	0.71 (1.37)	2.25 (1.85)		
Orgasm	Desirable performance (> 4/3)	8 (12.5)	25 (39.7)	0.001*	< 0.001***
	Poor performance (≤ 4/.3)	56 (87.5)	38 (60.3)		
	Total	64 (100.0)	63 (100.0)		
	Mean (SD)	0.76 (1.46)	2.27 (1.91)		
Satisfaction	Desirable performance (> 8/3)	4 (6.2)	16 (25.4)	0.003*	< 0.001***
	Poor performance (≤ 8/3)	60 (93.8)	47 (74.6)		
	Total	64 (100.0)	63 (100.0)		
	Mean (SD)	1.48 (1.17)	2.69 (l1.48)		
Sexual pain	Desirable performance (> 8/3)	4 (6.2)	16 (25.4)	0.003*	< 0.001***
	Poor performance (≤ 8/3)	60 (93.8)	47 (74.6)		
	Total	64 (100.0)	63 (100.0)		
	Mean (SD)	0.80 (1.48)	2.39 (1.94)		
Total score	Desirable performance (> 28)	0	2 (3.2)	0.496**	< 0.001***
	Poor performance (≤ 28)	64 (100.0)	61 (96.8)		
	Total	64 (100.0)	63 (100.0)		
	Mean (SD)	7.21 (6.40)	14.67 (9.10)		

*Chi-square test.

**Chi-square test based on Monte Carlo simulation.

***T-test of two independent groups.

In Table3 a multiple linear regression analysis was performed to examine the impact of colostomy on FSFI, controlling for demographic factors. The total score of the FSFI served as the dependent variable, while demographic variables were entered as independent variables. Univariate Linear Regression: Women with colostomy bags had a significantly lower average total FSFI score compared to the reference group (β = − 7.46, *p * < 0.001). Multivariate Linear Regression: After adjusting for other variables, women with colostomy bags showed a significantly lower average total FSFI score compared to the reference group (β = − 4.96, *p* < 0.001). These findings suggest that the presence of a colostomy bag has a substantial negative impact on FSFI, even when considering other participant’s’ characteristics.

**Table 3:** The application of univariate and multiple linear regression models in assessing the FSFI in patients with and without colostomy bag.

Variable	Classes	Univariate linear regression		Multiple linear regression	
		β (95% CI)	*P*-value	β (95% CI)	*P*-value
Group	With colostomy	− 7.46 (− 10.22, − 4.70)	< 001/0	− 4.96 (− 7.36, − 2.56)	< 001/0
	Without colostomy	Reference string		Reference string	

_R squared= .573 (Adjusted R squared= .502)_.

Table 4 displays the statistical analysis results of the IIEF questionnaire, assessing men's sexual performance in five domains. The analysis revealed no significant differences, both qualitatively and quantitatively, between men with and without colostomy bags in any of the domains (*p* < 0.005).

**Table 4:** Distribution of frequency, percentage, the mean and standard deviation of the international index of erectile function (IIEF) and comparing the scores separately for having and not having a colostomy bag (n = 129).

Variable	Classes (Cut of points)	Male with colostomy N (%)	Male without colostomyN (%)	*P*-value(Qualitative)	*P*-value(Standardized & Quantitative)
Erectile function	Severe disfunction (1–6)	19 (30.2)	20 (30.3)	0.328*	0.189***
	Moderate disfunction (7–12)	27 (42.9)	22 (33.3)		
	Mild to moderate disfunction (13–18)	9 (14.3)	14 (21.2)		
	Mild disfunction (19–24)	8 (12.7)	7 (10.6)		
	No disfunction (25–30)	0	3 (4.5)		
	Total	63 (100.0)	66 (100.0)		
	Mean (SD)	10.25 (5.89)	11.67 (6.24)		
Orgasm function	Severe disfunction (0–2)	28 (44.4)	28 (42.4)	0.178*	0.292***
	Moderate disfunction (3–4)	21 (33.3)	14 (21.2)		
	Mild to moderate disfunction (5–6)	7 (11.1)	16 (24.2)		
	Mild disfunction (7–8)	7 (11.1)	8 (12.1)		
	No disfunction (9–10)	0	0		
	Total	63 (100.0)	66 (100.0)		
	Mean (SD)	3.67 (1.90)	4.05 (2.15)		
Sexual desire	Severe disfunction (= 2)	28 (44.4)	28 (42.4)	0.178*	0.292***
	Moderate disfunction (3–4)	21 (33.3)	14 (21.2)		
	Mild to moderate disfunction (5–6)	7 (11.1)	16 (24.2)		
	Mild disfunction (7–8)	7 (11.1)	8 (12.1)		
	No disfunction (9–10)	0	0		
	Total	63 (100.0)	66 (100.0)		
	Mean (SD)	3.67 (1.90)	4.05 (2.15)		
Satisfaction with sexual contact	Severe disfunction (0–3)	32 (50.8)	32 (48.5)	0.263**	0.413***
	Moderate disfunction (4–6)	8 (12.7)	3 (4.5)		
	Mild to moderate disfunction (7–9)	10 (15.9)	15 (22.7)		
	Mild disfunction (10–12)	13 (20.6)	14 (21.2)		
	No disfunction (13–15)	0	2 (3.0)		
	Total	63 (100.0)	66 (100.0)		
	Mean (SD)	4.25 (4.75)	4.97 (5.11)		
Comprehensive satisfaction	Severe disfunction (= 2)	23 (36.5)	30 (45.5)	0.076*	0.973***
	Moderate disfunction (3–4)	23 (36.5)	12 (18.2)		
	Mild to moderate disfunction (5–6)	5 (7.9)	9 (13.6)		
	Mild disfunction (7–8)	6 (9.5)	12 (18.2)		
	No disfunction (9–10)	6 (9.5)	3 (4.5)		
	Total	63 (100.0)	66 (100.0)		
	Mean (SD)	4.33 (2.48)	4.32 (2.53)		
Total score	Severe disfunction (6–20)	34 (54.0)	29 (43.9)	0.207*	0.318***
	Moderate disfunction (21–32)	11 (17.5)	10 (15.2)		
	Mild to moderate disfunction (33–45)	8 (12.7)	11 (16.7)		
	Mild disfunction (46–58)	10 (15.9)	11 (16.7)		
	No disfunction (59–75)	0	5 (7.6)		
	Total	63 (100.0)	66 (100.0)		
	Mean (SD)	26.17 (15.30)	29.05 (17.14)		

*Chi-square test.

**Chi-square test based on Monte Carlo simulation.

***T-test of two independent groups.

Table 5 shows the scores obtained by male samples, which are standardized in all domains as well as the total score, which shows the scores separately for with and without a colostomy bag.

**Table 5:** Distribution of raw and standardized scores of the International Index of Erectile Function.

Variable	Number of Items	Minimum for the score	Maximum for the score	Mean (SD)		95% confidence interval		Standardized mean (standard deviation)	
				With colostomy	Without colostomy	With colostomy	Without colostomy	With colostomy	Without colostomy
Erectile function	6	0	30	10.25 (5.89)	11.67 (6.24)	8.77–11.74	10.13–13.20	34.18 (19.64)	38.89 (20.81)
Orgasm function	2	0	10	3.67 (1.90)	4.05 (2.15)	3.19–4.15	3.52–4.57	36.67 (19.00)	40.45 (21.51)
Sexual desire	2	2	10	3.67 (1.90)	4.05 (2.15)	3.19–4.15	3.52–4.57	20.83 (23.76)	25.57 (26.89)
Satisfaction with sexual contact	2	0	15	4.25 (4.75)	4.97 (5.11)	3.06–5.45	3.71–6.23	28.36 (31.70)	33.13 (34.11)
Comprehensive satisfaction	2	2	10	4.33 (2.48)	4.32 (2.53)	3.71–4.96	3.70–4.94	29.17 (31.10)	28.98 (31.63)
Total Score	15	4	75	26.17 (15.30)	29.05 (17.14)	22.32–30.03	24.83–33.26	31.23 (21.56)	35.28 (24.14)

In Table6 a multiple linear regression analysis was performed to examine the impact of colostomy on IIEF, controlling for demographic factors. The total score of the IIEF served as the dependent variable, while demographic variables were entered as independent variables. Univariate Linear Regression: Men with colostomy bags had a nonsignificant lower average total IIEF score compared to the reference group (β = − 4.04, p = 0.318). Multivariate Linear Regression: After adjusting for other variables, men with colostomy bags showed a significantly lower average total IIEF score compared to the reference group (β = − 9.32, p = 0.023).

**Table 6:** The application of univariate and multiple linear regression models in assessing the IIEF in patients with and without colostomy bag.

Variable	Classes	Univariate linear regression		Multiple linear regression	
		β (95% CI)	*P*-value	β (95% CI)	*P*-value
Group	With colostomy	− 4.04 (− 12.03, 3.94)	0.318	− 9.32 (− 17.32, − 1.33)	0.023
	Without colostomy	Reference string		Reference string	

_R squared=_ ._437 (Adjusted R squared= .345)_.

**Figure 2:**
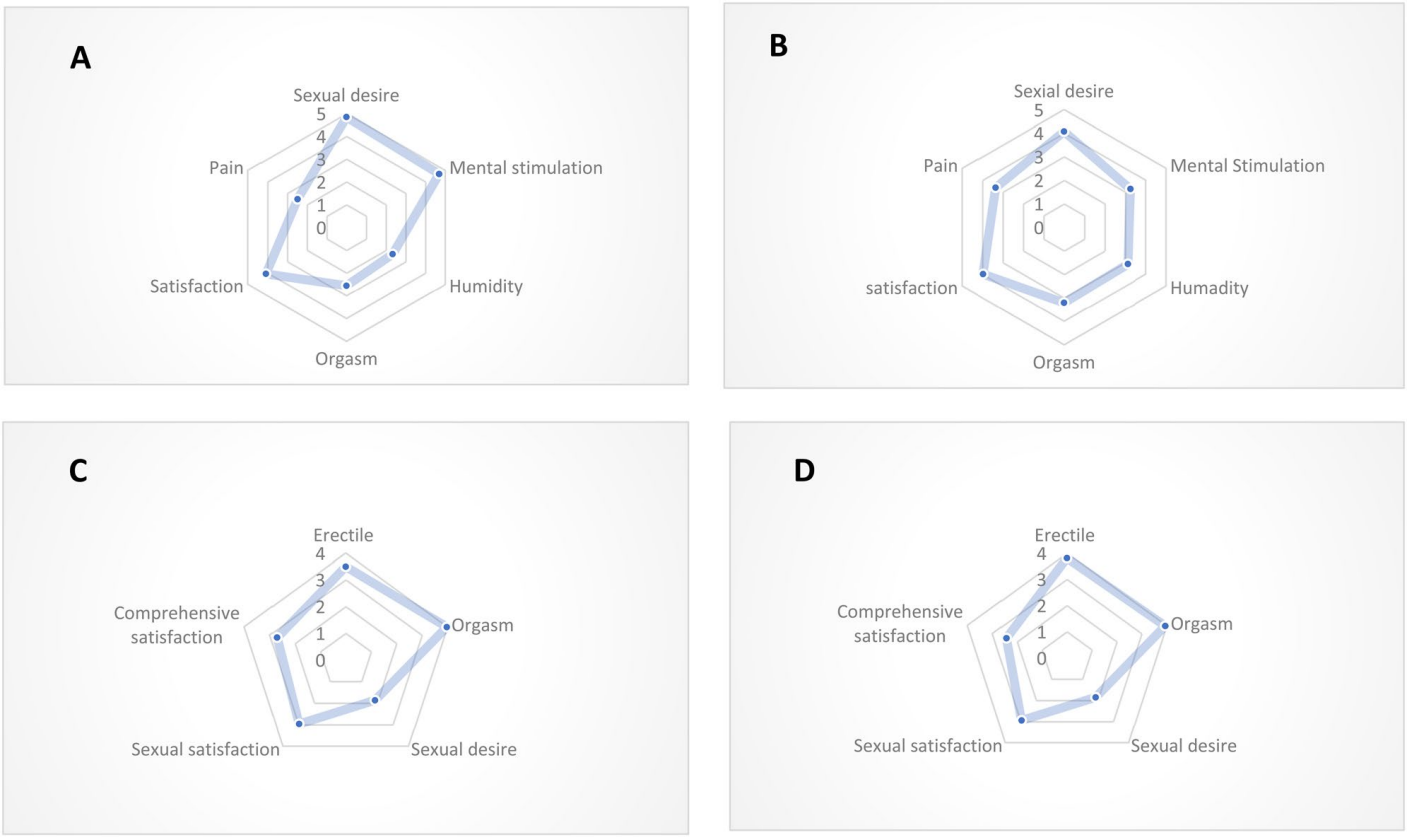
Radar diagram of IIEF and FSFI scores in different domains.

Figure 2 demonstrating mean rank values that the statistical analysis was performed using the Friedman test, and mean ranks were extracted to assess differences among the groups. Chart A depicts the FSFI performance in patients with colostomy, while Chart B represents the performance in patients without colostomy. Similarly, Chart C displays the IIEF performance in patients with colostomy, and Chart D shows the performance in patients without colostomy. These radar charts visually illustrate the differences in sexual function outcomes based on colostomy status.

## Discussion

One of the notable findings in this study was the overall low sexual performance scores observed in both men and women with colorectal cancer, irrespective of the presence of a colostomy bag. Previous research has primarily focused on the surgical aspects of ostomies, with limited attention given to the psychological and psycho-emotional effects associated with sexual function^30–34^. Recognizing the importance of sexual health in individuals with colorectal cancer, as highlighted by previous studies^35^, this study further reinforces the significance of addressing this issue.

While comparing sexual performance between men and women with colorectal cancer, the absence of a statistically significant difference among men with and without colostomy bags is an important finding. Both groups exhibited lower levels of sexual performance, indicating a need for support and intervention. On the other hand, a significant difference was observed among women, with those with colostomy bags experiencing lower sexual performance scores compared to those without. Specifically, aspects of satisfaction and sexual pain showed statistical disparities. These findings emphasize the importance of providing comprehensive support to women in all dimensions of sexual well-being.

Time plays a critical role in the successful adaptation to life with an ostomy. Over time, many challenges such as body image changes, concerns about ostomy bag leakage, odor, bowel sounds, and loss of libido tend to diminish^36^. Given these observations, it is crucial to provide timely supportive interventions immediately after ostomy surgery, as suggested by previous studies^32^ The present study underscores the need for early post-surgery intervention, highlighting the importance of dedicated care and attention to address these concerns promptly.

Research on cancer patients consistently demonstrates the relationship between disease severity, psychological distress, and overall quality of life^37^. Sexual health, as defined by the World Health Organization (WHO), is a fundamental aspect of individual, couple, and family well-being, as well as societal and economic development^38^. Sexual activity and performance play a pivotal role in overall quality of life. Unfortunately, upon cancer diagnosis, patients often prioritize anti-cancer treatments and tend to overlook the sexual dimension, considering it a neglected aspect^35, 39^. It is noteworthy that previous studies have found that sexually active older adults report greater life satisfaction and better overall health compared to those who are sexually inactive^40^. By improving sexual performance, we not only enhance an essential aspect of individuals' lives but also indirectly contribute to their overall quality of life.

The present findings underscore the paramount importance of identifying and addressing sexual concerns among colon cancer patients early on in order to mitigate the adverse effects on intimate relationships and overall well-being. Previous research has indicated a significant disparity between men and women regarding their willingness to discuss sexual issues, with women being notably less likely to openly express their concerns about sexual problems Furthermore, the study revealed a noteworthy distinction between women with and without a colostomy in terms of sexual performance, whereas no significant difference was observed among men. These results emphasize the need for targeted interventions aimed at addressing the sexual concerns of women undergoing cancer treatment, while recognizing that some studies suggest a gender-related association with sexual dysfunction in cancer patients, while others do not^20, 32, 41, 42^, Therefore, a comprehensive evaluation of patients is warranted, taking into account various factors such as gender, age, partner status, and treatment status, regardless of the specific findings of this study.

Married women facing the challenges of a colostomy may harbor fears of partner rejection and encounter difficulties in maintaining their relationship as both partners navigate the adjustments. Numerous qualitative studies consistently highlight the significant role played by the acceptance or rejection of a life partner in shaping a woman's acceptance of the stoma and her ability to cope with sexual problems and intimate relationships^41, 43^. Thus, when delivering support services and designing intervention programs to enhance the quality of sexual life, it is imperative to adopt a holistic approach that encompasses the needs of both the patient and their partner, rather than focusing solely on the individual.

## Conclussion

In conclusion, this study highlights the importance of addressing sexual concerns in individuals with colorectal cancer, regardless of the presence of a colostomy bag. Both men and women exhibited low sexual performance scores, emphasizing the need for comprehensive support and interventions in this patient population. Among men, no significant differences were found between those with and without colostomy bags. However, both groups showed lower levels of sexual performance, indicating the importance of support and intervention for men with colorectal cancer. For women, a significant disparity was observed, with those having colostomy bags experiencing lower sexual performance scores compared to those without. Specific areas such as satisfaction and sexual pain were particularly affected. Targeted support services are crucial to address the unique sexual concerns faced by women undergoing cancer treatment, especially those with colostomies.

Early implementation of supportive interventions following ostomy surgery is vital for successful adaptation. Challenges such as body image changes, leakage concerns, odor, bowel sounds, and loss of libido tend to diminish over time. Prompt post-surgery interventions should be provided to address these concerns and promote overall well-being. Identifying and managing sexual concerns in colon cancer patients early on is essential to minimize the adverse effects on intimate relationships and overall well-being. Tailored interventions are needed to address the specific sexual concerns of female patients, considering factors such as gender, age, partner status, and treatment status. Married women with colostomies may face fear of partner rejection and relationship difficulties. The acceptance or rejection of a life partner significantly influences a woman's acceptance of a stoma and her ability to cope with sexual problems and intimate relationships. Support services and intervention programs should adopt a holistic approach that includes the needs of both the patient and their partner.

### Limitations of the study

The present study acknowledges several limitations that should be taken into account. Firstly, the small sample size restricts the generalizability of the findings. It is important to note that a larger and more diverse sample would be beneficial for drawing broader conclusions. Secondly, the reliance on self-reported measures introduces the potential for biases and inaccuracies in recall, which may affect the validity of the results. Another limitation is the cross-sectional design employed in this study, which prevents the establishment of causality and temporal relationships. A longitudinal design could provide more robust evidence and allow for the examination of changes over time. Furthermore, the study's focus on a specific colorectal cancer population limits its applicability to other cancer types and general populations. Additionally, the study did not explore important factors such as psychological variables and treatment modalities, which may have an impact on sexual function. Moreover, a significant limitation of this study is the lack of differentiation between colon and rectal cancer. These two types of cancer have distinct impacts on sexual dysfunction, and analyzing them separately would yield more specific and meaningful results. Lastly, the absence of pre-treatment assessment of sexual function is a notable limitation. Evaluating sexual function prior to treatment would have provided a baseline measure and a better understanding of the effects of cancer and interventions.

### Recommendations for further studies

Based on the study's limitations, the following recommendations for future research can be made: 1—Increase sample size: Conduct larger and more diverse studies to enhance generalizability of findings. 2—Utilize objective measures: Incorporate validated questionnaires or clinical evaluations to obtain more accurate data on sexual function. 3—Employ longitudinal design: Follow individuals over time to examine causality and changes in sexual function throughout the cancer journey. 4—Differentiate cancer types: Investigate the specific impact of colon and rectal cancer on sexual performance separately. 5—Explore psychological factors: Assess the influence of psychological variables such as body image, self-esteem, anxiety, and depression on sexual function. 6—Consider treatment modalities: Investigate the effects of different treatment approaches (e.g., surgery, chemotherapy, radiation) on sexual performance. 7—Examine cultural influences: Conduct research in diverse cultural contexts to understand the role of cultural factors in sexual performance. 8—Incorporate partner perspectives: Include the perspectives of partners and address the dynamics of intimate relationships in future studies.

By addressing these recommendations, future research can provide a more comprehensive understanding of sexual performance in colorectal cancer patients and inform the development of targeted interventions to improve sexual well-being.

## Supplementary Information

Supplementary Information 1.

Supplementary Information 2.

Supplementary Information 3.

## Data Availability

Data of this manuscript has been gathered through questionnaires by patients themselves the questioner was actively present when the questionnaires were filled and provided the necessary guidance in filling the questionnaires by the patients. The data sets used and analyzed for the current study are available upon reasonable request of the corresponding author Dr. Mohammadhassan Sahebihagh (sahebihagh@yahoo.com). All raw data in the Excel files have been uploaded to the supplementary material section.
