# Supplementary material for: Comparison of sexual function of people with colorectal cancer with and without colostomy bag in Iran: a comparative cross-sectional study
Source: Sci Rep. 2023 Aug 2;13:12558. doi: 10.1038/s41598-023-39728-9 (PMC10397230; doi:10.1038/s41598-023-39728-9)
Supplement: Supplementary file 1 — Supplementary Information 1. [file 41598_2023_39728_MOESM1_ESM.pdf]

Tabriz University of Medical Sciences

Research Ethics Committees Certificate

Approval ID:	IR.TBZMED.REC.1401.046	Approval Date:	2022-04-06
Evaluated by:	Research Ethics Committees of Tabriz University of Medical Sciences		
Status:	Approved		
Approval Statement:	The project was found to be in accordance to the ethical principles and the national norms and standards for conducting Medical Research in Iran.<br>Notice:<br>1. Although the proposal has been approved by the Biomedical Research Ethics Committee, meeting the professional and legal requirements is the sole responsibility of the PI and other project collaborators.<br>2. This certificate is reliant on the proposal/documents received by this committee on 2022-04-06. The committee must be notified by the PI as soon as the proposal/documents are modified.		
Thesis Title:	`Comparison of quality of life and sexual function at colorectal cancer's patients with and without colostomy bag in Tabriz hospitals 2022`		
Supervisor:	Name: mohammad Hasan sahebi hagh<br>Email: sahebihagh@tbzmed.ac.ir		
Student:	Name: amirmohammad dahouri<br>Email: amirmohammaddahouri61@gmail.com		

Dr. Bahman Naghipour
Committee Director
Tabriz University of Medical Sciences

Dr. Parviz Shahabi
Committee Secretary
Tabriz University of Medical Sciences
